# Medical History from the Earliest Times

**Published:** 1893-04-15

**Authors:** E. T. Withington


### Medical History from the Earliest Times.

By E. T. Withington, M.A., M.B. Oxon.

### L.—THE GREAT ANATOMISTS.

Though the intellectual revival, which, during the fifteenth and sixteenth centuries, produced such great results in art, literature, and religion was slow in effecting a revolution in medicine, the activity in other directions had important influences on the healing art. We have already seen how the study of classical literature gave rise to a Greek, as opposed to the dominant Greeco-Arabic, school of physicians, whose controversies lasted more than a century, and how the rejection of authority in religion coincided with the appearance of medical anarchists and freethinkers, of whom Paracelsus was the chief. The renaissance of art was not without stimulating effects upon another form of activity in medicine, less noisy and conspicuous, but destined to produce far greater and more permanent results.

Two centuries had elapsed since Mondino of Bologna "restored" anatomy, but hardly any progress had been made. Bodies were opened rather than dissected, and little more was done than to point out how the position of the viscera corresponded with the descriptions of Galen. The requirements of the great artists, Leonardo-da-Vinci, Raphael, Michael Angelo, demanded something more than this, and when, in 1521, Berengar of Carpi, Professor at Bologna, published a commentary on his predecessor's work, intended for artists as well as physicians, and calling in question assertions not only of Mondino, but of Galen himself, he inaugurated a new era—that of the great anatomists. Space will not permit the enumeration of the various discoveries attributed to each of the distinguished men to be now briefly mentioned, nor are such lists of much historical value; the two facts important to notice are the gradual undermining of the Galenic infallibility, and the anatomical preparation for Harvey's great discovery. Berengar did something in this direction by carefully describing the structure and valves of the heart; but the real beginner of the work was Andrew Vesalius, born at Brussels, 1514; professor at Padua 1537-46, while he taught also at Bologna and Pisa; died 1564, shipwrecked on the island of Zante. Why is Vesalius the greatest of anatomists? Not because of the facts he discovered, for in these he was equalled, if not surpassed, by Eustachius and Fallopius; but because he first saw that it was useless to continue commenting upon or correcting Galen and Mondino, and that anatomy must begin afresh; and because he made this fresh beginning, as well as any single man could make it, in his immortal work "On the Structure of the Human Body," published 1543, when he was only twenty-nine years old. The importance of this work is best seen in the attacks of the Galenists, especially of his master, Sylvius, who called him "a madman (vesanus), whose pestilential breath poisons Europe"; in the admiration of such men as Fallopius and Paré; and, above all, in the impulse which it gave to the study of anatomy. A few years after its publication the professional chairs of the Universities of Italy were filled with men whose names are daily repeated in every dissecting room. Vesalius, indeed, had gone, leaving his work at Padua to his prosector Columbus, who declared that there was more to be learnt from dissecting, and especially from vivisecting one animal, than from reading all the anatomy of Galen or feeling pulses for months, and whose zeal was rewarded by the discovery of the pulmonary circulation. The ancient fame of Bologna was well supported by Arantius and Varolius. Vidius had returned from establishing the anatomical school at Paris to teach at Pisa. Eustachius was at Rome, Ingrassias at Naples, and in 1551 Columbus resigned his chair at Padua to one who, in the beauty of his personal character as well as in the amount and excellence of the work accomplished during his short life, stands first of Italian anatomists—Gabriel Fallopius (1523-62).

The state of the science in the Teutonic countries presents a marked contrast to the above. In Germany, where Paracelsus is supposed to have reformed medicine, professors continued to lecture in the old style of Galen and Avicenna, to students hardly more numerous than themselves. England had for the first time produced in Linacre and Caius medical teachers of European reputation, and the anatomical work of Vicary, though from a wider standpoint it can hardly be considered more than a parody on the new science, was, at least, a promise of greater things to come. But the only country which could in any way compare with Italy was France. When the University of Paris was remodelled in 1542, they sent for Guido Guidi from Florence to organise the medical faculty, and he did so with a rapidity and success which caused the wits of the age to declare that "Vidus Vidius venit, vidit, vicit." He left the continuation of his work to Jacques Dubois (Sylvius) of Amiens, a teacher of men greater than himself—Vesalius, Servetus, Etienne—to whom, however, though the friend of Galen rather than of truth, anatomy is indebted for much of its nomenclature. He chiefly dissected animals, perhaps from the difficulty of obtaining human subjects, which compelled Vesalius to rob the gallows, and the gentle and pious Rondelet to inaugurate his anatomical theatre at Montpellier by dissecting the body of one of his own children.

It was Sylvius who seems to have made what might have been the most important of anatomical discoveries, that of the valves in the veins, for as early as 1536 (?) he noiced the existence of what he calls "epiphyses" in the vena azygos, the jugular, brachial, and other vessels, and years later the valve in the vena azygos was again seen by Canani, professor at Ferrara. But these notices attracted so little attention that when Fabricius rediscovered the valves in 1574, he could assert that no anatomist, ancient or modern, had ever mentioned them. He published his treatise, "De Venarum Ostiolis," in 1603, with beautiful illustrations of the valves, and declares that they serve, like the locks and weirs on a river, to prevent the too rapid flow of the nutritive, fluid and to ensure its equable distribution. Etienne, the pupil of Sylvius, had already (1545) described valves in the hepatic veins (!) and had given the true explanation of their function to prevent the reflux of the blood but the discovery did not lead him any further, for even the Galenists admitted that the flow in the hepatic veins is towards the heart.

It is interesting to note that the three most distinguished pupils of the great orthodox anatomist were accused of heresy in religion as well as science. Vesalius was persecuted by the theologians, Etienne perished in the dungeons of the Inquisition, while Servetus, as all know, met a yet more terrible fate at the hands of Calvin. But the man who nearly anticipated Harvey deserves more than a mere mention. Medical history first meets with Michael Servetus, M.A., M.B. Paris, on a February afternoon, 1538, in the anatomy theatre of that University, where he has just finished dissecting a human subject, and is disputing with the Dean of the Medical Faculty about some astrological lectures he had been giving. A few days later he sends "certain Italian friends" to apologise to the Dean. It may have been in dissecting this subject, a rare thing at that time, that he noticed the solidity of the cardiac septum and large size of the pulmonary artery, which led him to the discovery of the so-called lesser circulation, and the "Italian friends" may have taken some hint of the new theory to Columbus at Padua. At any rate the first description of the pulmonary circulation was published by Servetus in his "Restitution of Christianity," 1553, and the same theory was contained in the MS. copy of the work which he sent to Calvin at the end of 1545 or beginning of 1546. The Reformer refused to return the manuscript, and lay in wait for seven years to slay its author, but a partial copy exists at Paris and contains the famous passage on the "vital spirits." Servetus begins by rejecting the doctrine of three spirits—natural, vital, and animal—residing in the veins, arteries, and nerves respectively, and declares that the natural and vital spirits are not distinct, and that the fluids in the veins and arteries are of the same nature, thus removing one of the greatest hindrances in the way of Harvey's discovery. The vital spirit (*i.e.*, arterial blood) is formed from "a mixture made in the lungs of the inbreathed air with the blood which the right ventricle communicates to the left. This takes place, not through the cardiac septum as generally believed, but by consummate art the subtle blood of the right ventricle is moved in a long passage through the lungs; by them it is prepared; it is made bright; it is transfused from the pulmonary artery to the pulmonary vein . . . it is purged from fume by expiration." He confirms this by the arguments noticed above, compares the connection between the artery and vein in the lungs with that between the portal and hepatic veins in the liver, and says that in the transition from artery to vein "there is a new kind of vessels in the lung formed out of vein and artery." It would be difficult to add much to this even at the present day, and Servetus might well claim to have discovered something unknown to Galen or to the greatest philosophers.

